# New Approaches of PARP-1 Inhibitors in Human Lung Cancer Cells and Cancer Stem-Like Cells by Some Selected Anthraquinone-Derived Small Molecules

**DOI:** 10.1371/journal.pone.0056284

**Published:** 2013-02-25

**Authors:** Yu-Ru Lee, Dah-Shyong Yu, Ya-Chun Liang, Kuo-Feng Huang, Shih-Jie Chou, Tsung-Chih Chen, Chia-Chung Lee, Chun-Liang Chen, Shih-Hwa Chiou, Hsu-Shan Huang

**Affiliations:** 1 Graduate Institute of Life Science, National Defense Medical Center, Taipei, Taiwan; 2 Graduate Institute of Medical Sciences, National Defense Medical Center, Taipei, Taiwan; 3 School of Pharmacy, National Defense Medical Center, Taipei, Taiwan; 4 Uro-Oncology Laboratory, Division of Urology, Department of Surgery, Tri-Service General Hospital, Taipei, Taiwan; 5 Department of Pharmacy Practice, Tri-Service General Hospital, Taipei, Taiwan; 6 Chi-Mei Medical Center, Tainan, Taiwan; 7 Institute of Pharmacology, National Yang- Ming University, Taipei, Taiwan; 8 Department of Medical Research and Education, Taipei Veterans General Hospital, Taipei, Taiwan; University of Pecs Medical School, Hungary

## Abstract

Poly (ADP-ribose) polymerase-1 (PARP-1) and telomerase, as well as DNA damage response pathways are targets for anticancer drug development, and specific inhibitors are currently under clinical investigation. The purpose of this work is to evaluate anticancer activities of anthraquinone-derived tricyclic and tetracyclic small molecules and their structure-activity relationships with PARP-1 inhibition in non-small cell lung cancer (NSCLC) and NSCLC-overexpressing Oct4 and Nanog clone, which show high-expression of PARP-1 and more resistance to anticancer drug. We applied our library selected compounds to NCI's 60 human cancer cell-lines (NCI-60) in order to generate systematic profiling data. Based on our analysis, it is hypothesized that these drugs might be, directly and indirectly, target components to induce mitochondrial permeability transition and the release of pro-apoptotic factors as potential anti-NSCLC or PARP inhibitor candidates. Altogether, the most active **NSC747854** showed its cytotoxicity and dose-dependent PARP inhibitory manner, thus it emerges as a promising structure for anti-cancer therapy with no significant negative influence on normal cells. Our studies present evidence that telomere maintenance should be taken into consideration in efforts not only to overcome drug resistance, but also to optimize the use of telomere-based therapeutics. These findings will be of great value to facilitate structure-based design of selective PARP inhibitors, in general, and telomerase inhibitors, in particular. Together, the data presented here expand our insight into the PARP inhibitors and support the resource-demanding lead optimization of structurally related small molecules for human cancer therapy.

## Introduction

Lung cancers are generally categorized as small cell lung carcinomas (SCLC) and non-small cell lung carcinomas (NSCLC), which are further subclassified as adenocarcinoma (AC), squamous cell carcinoma (SCC), and large cell carcinoma (LCC) [1], [2]. Cancer stem cells are small reservoirs of self-sustaining cells with the exclusive ability for self-renewal and tumor maintenance [3]. Although new chemotherapy agents and radiotherapy have improved patients' survival and quality of life, the long-term survival rate of patients with lung AC remains unsatisfactory. Chemotherapy in the last decade has been used mainly for palliation rather than reduction in mortality; there is still an urgent need for further searching the novel small molecules for new chemotherapy agents.

PARP, which plays a role in the repair of single-stranded DNA (ssDNA) breaks, has a number of distinct biochemical activities which have been an attractive target for the design of anticancer agents [4]–[6]. Over the past decade, many small molecules with inhibition of PARP family have been synthesized and some of them are currently being tested in clinical trials as cancer therapies [7]–[9]. Although they have been studied for their utility in DNA damage detection and repair, the extent to which PARP control other specific developmental process is not clear. The development of specific, potent, effective, and safe PARP inhibitors has become an area of active research and much recent publication in the PARP field. For this reason, inhibiting PARP activity, especially PARP-1, with small molecules reduces repair of ssDNA breaks, and is likely to be useful for treating cancers, stress, inflammatory responses and cardiovascular disease [7], [8]. Clinical trials which are now underway are examining the safety and efficacy of PARP-1 inhibitors as anti-cancers, including breast, uterine, and ovarian cancers [9]. In addition, the functions of PARP in DNA damage responses and protection of telomeres may overlap with the telomerase [10]. A previous report suggested that telomerase and PARP play a role in chromosome instability and DNA damage [11]. In many cases, the efficacy of the inhibitors may be due to the synthetic lethality between PARP inhibition and a genetic lesion in the tumor cells [12]–[14].

**Figure 1:**
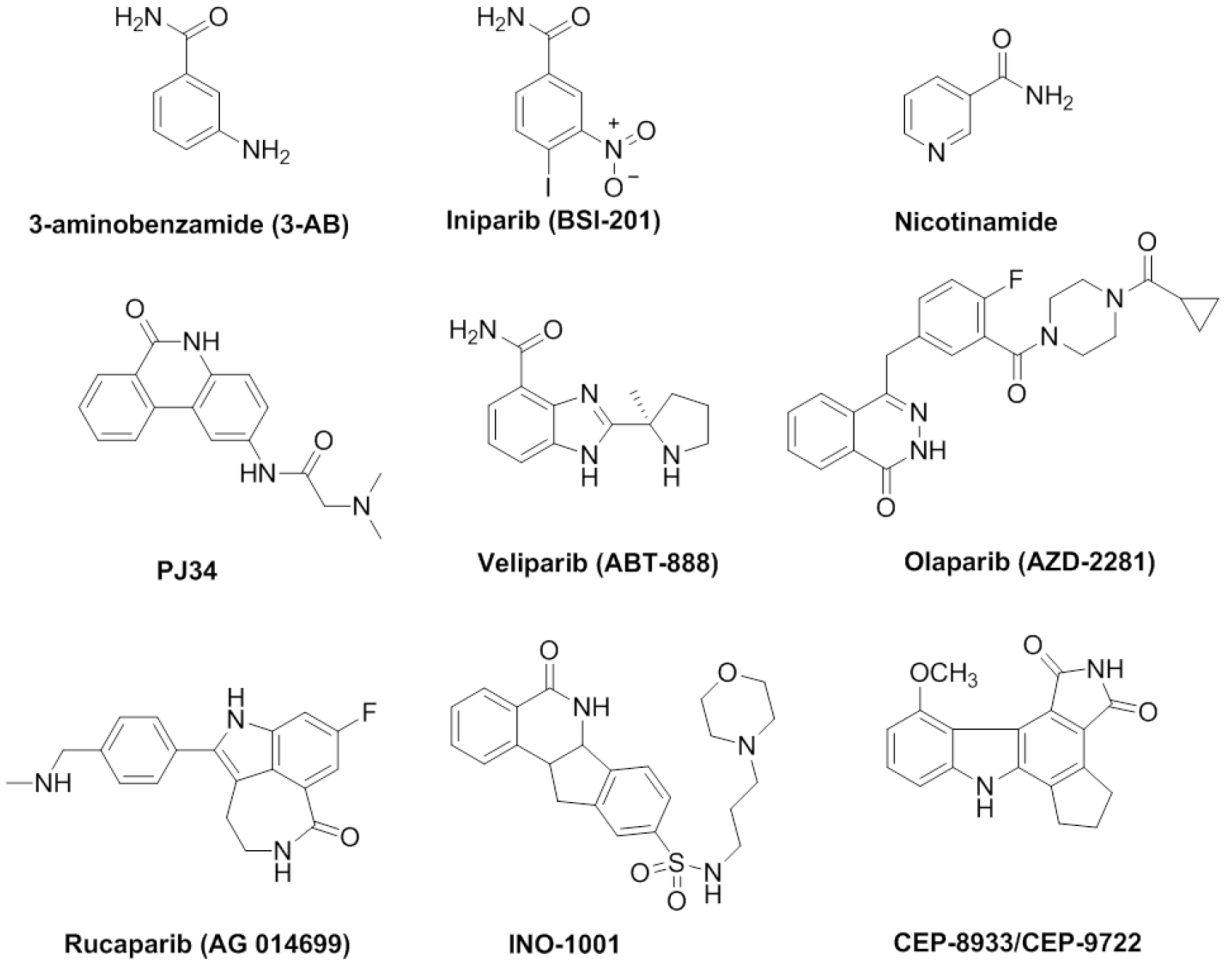
Investigational structures of PARP-1 Inhibitors.

3-Aminobenzamide (3-AB) is a first generation PARP-1 inhibitor [15], [16], but it lacks the requisite selectivity and potency to be useful in clinics or as a research tool [9], [17]. Furthermore, nicotinamide, the smaller cleavage product of NAD^+^ also exerts inhibitory effect on PARP-1 [18]. Veliparib (ABT-888) is also a novel and potential anti-cancer drug acting as a PARP-1 inhibitor [19]. Olaparib (AZD2281) has shown promising clinical efficacy in nonrandomized phase II trials in patients with ovarian cancer with BRCA1 or BRCA2 deficiency [20]. Iniparib (BSI-201) is notable for its simple structure, but it kills normal and neoplastic cells at high concentrations and should not be considered as PARP inhibitor [21]. Other drugs such as INO-1001, CEP-8933/CEP-9722 and phenanthrene-related derivatives PJ-34 have also been evaluated in clinical trials so far [22]. PJ-34 was also the most potent compound in this field [23]. We provide PARP-1 activities of some selected compounds, as well as an in-depth investigation of our papers published or unpublished in the past few years that have provided new insights into the inhibition of PARP-1 in the nucleus (Figure 1) [24]–[33].

Toward supporting the aforementioned hypothesis, studies have shown that p53-deficient breast cancer cells treated with a PARP-1 inhibitor lose resistance to doxorubicin, a clinically active antitumor anthracycline antibiotic that promotes apoptosis [34]. Furthermore, the study show that phenanthrenes-related PARP-1 inhibitors have potent cytoprotective effects *in vitro* and *in vivo* [35].Another study showed that Cisplatin concentration can be reduced after being followed by treatment with PARP-1 inhibitor, PJ-34, to obtain the same cytotoxic effect [36]. As discussed in the article, targeting the base excision repair pathway with potent PARP-1 inhibitors is proving to be fruitful route to developing novel agents that may have activity not only as chemo- or radio-potentiating agents, but also as an active agent in DNA repair of deficient inherited cancers [37]. In this study, we highlight emerging information about the inhibition of PARP-1 in anthraquinone-derived derivatives outcomes, its interplay with antitumor activity against in the full panel of human tumor cell lines and the inhibition of these derivatives upon PARP-1. These and other related clinical discoveries have moved PARP-1 from interesting subjects of molecular analyses to the forefront as clinical targets for cancer treatment. In our approach to this research, we investigated newly discovered PARP inhibitors, the suitably modified anthraquinone-derived small molecules, to determine their effects on cell viability, western blot assay, cancer cells and their PARP-1 inhibition.

## Results and Discussion

### Antiproliferative evaluation of the selected small molecules

A previous report suggested that rhein could induce apoptosis in human promyelocytic leukemia cells (HL-60), characterized by caspase activation, PARP cleavage, and DNA fragmentation [38]. As we come to understand the links between the epigenetic status of telomeres, PARP and small-molecules will open new avenues for our understanding of new drugs development for cancer therapy. Understanding of the mode of action of anthraquinone-based anticancer drugs has increased in recent years, but still remains incomplete. In recent years, we also have harnessed a number of families of the telomere-targeted of anthraquinone-based pharmacophore to generate structural novelty and diversity for their biologically relevant studies. It was shown that the planar anthraquinones bind onto the end of the G-quadruplex structure through π–π stacking interactions with guanine residues [39]–[43]. It was also shown that amido-anthraquinones represent one of the best small molecules to modulate DNA duplexes and quadruplexes selectivity [44], [45].

**Figure 2:**
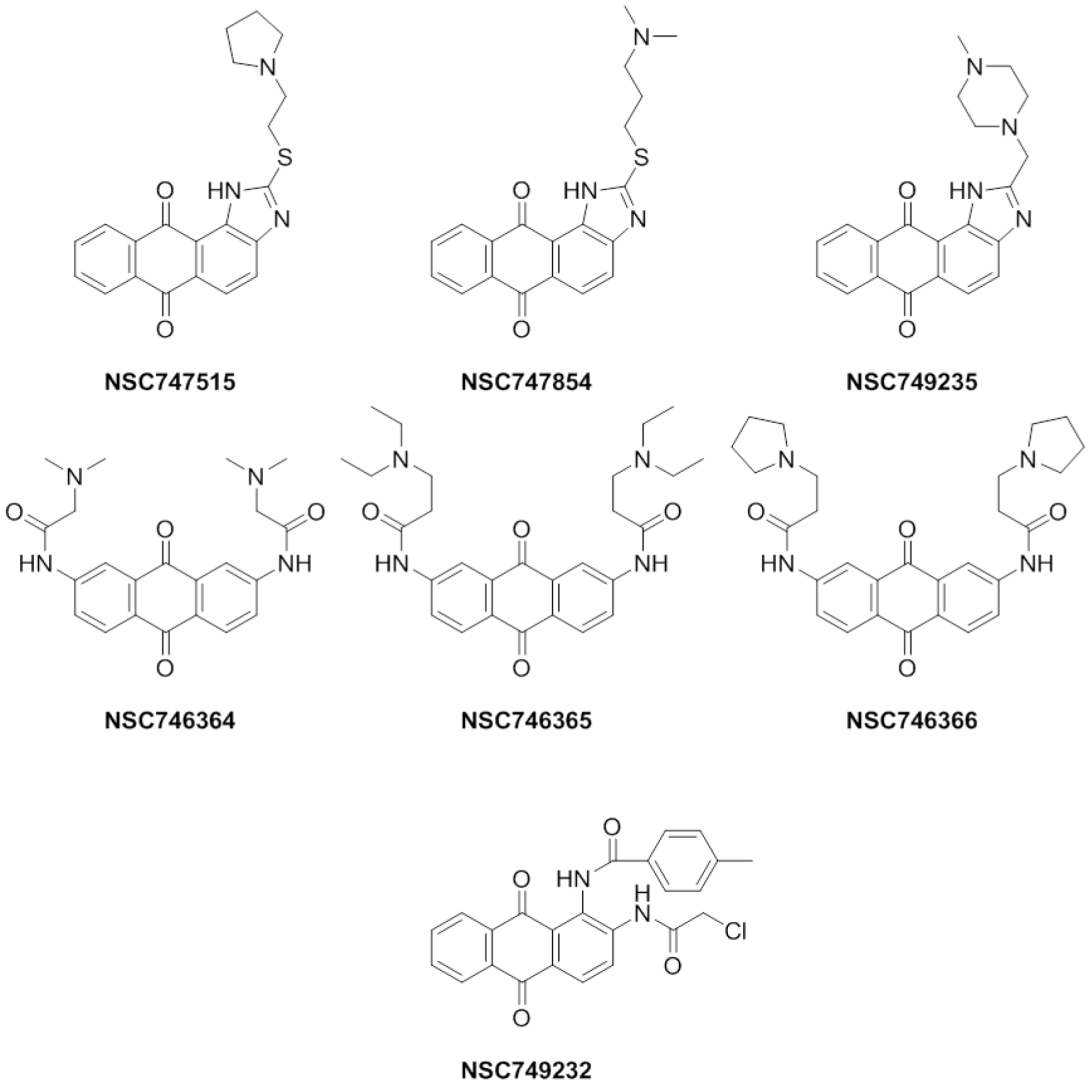
The anticancer drugs in use and seven promising putative PARP-1 inhibitor structures were under our research development [26],[27],[62]

**Figure 3:**
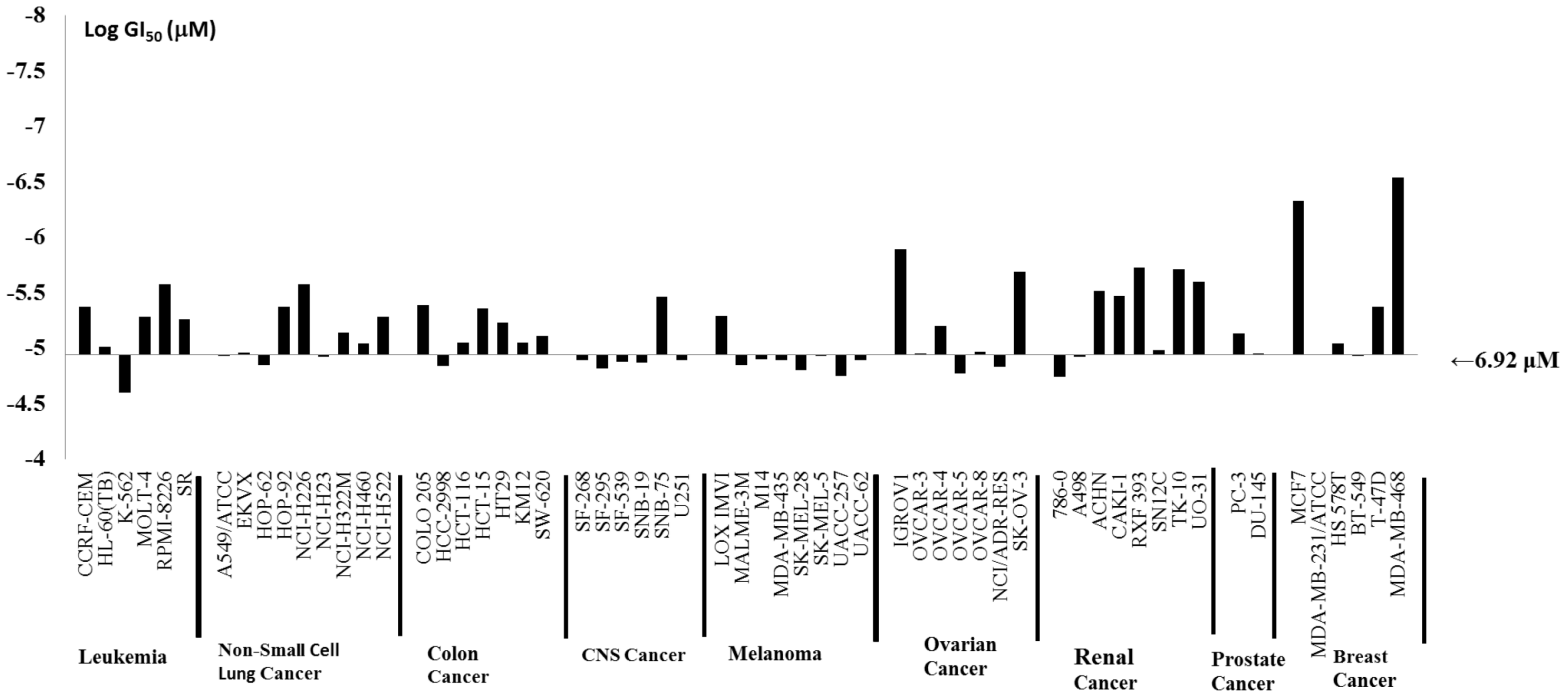
Antitumor activity of NSC747854. **a) A structure of NSC747854.** b) Antitumor activity of **NSC747854** in NCI-60 cancer cell lines which was submitted to the NCI Developmental Therapeutics Program (National Institutes of Health) in order to test its effect on a panel of 60 cancer cell lines derived from various tissues and organs. The GI_50_ at logarithmic scale were calculated and are shown in the bar graph. The middle line represents the median 50% growth inhibitory concentration for **NSC747854** (*i.e.* 6.92 µM).

The seven anthraquinone-derived small molecules (**NSC746364**, **NSC746365**, **NSC746366, NSC747515, NSC747854, NSC749235**, and **NSC749232**) (Figure 2) evaluated herein were selected because they exhibited significant cytotoxicity, which elicit activity against the full NCI's 60 panel of human tumor cell lines derived from nine cancer cell types: NSCLC, colon cancer, breast cancer, ovarian cancer, leukemia, renal cancer, melanoma, prostate cancer, and CNS cancer. There is additional evidence that these dose-dependent inhibited proliferations in all 60 cancer cell lines, cytotoxicity, and PARP-1 were derived from experiments shown in this study. Furthermore, all of these seven compounds were selected for further evaluation at five dose level screening. In this study they all show good activity with GI_50_, TGI and LC_50_ in NSCLC cell lines, respectively. Based on their NCI's analysis, it is hypothesized that these drugs might have potential to be anticancer drugs candidates. Toward this end, we chose NSCLC as our primary focus. Even **NSC747854** is more sensitive to the breast, renal, and ovarian cancers of NCI's 60 cell line screen (Figure 3). By taking advantage of recent structural insight into the NSCLC cell lines from the NCI Drug Screen Program, we analyzed the GI_50_, TGI, and LC_50_, as well as PARP inhibitory activity and the IC_50_ in lung cancer cell-overexpressing Oct4, Nanog clone (A3), lung cancer cell line (A549) and normal fibroblast cell line (HEL299), respectively. Our previous result suggested that the A549-overexpressing Oct4 and Nanog clone is more resistant to the anticancer drug, Cisplatin, as compared to the A549 parental cell [46].

The investigation covered advances made in our groups and provided discussions on some selected anthraquinone-derived small molecules which under various headings cover the structure-activity relationships (SARs), biological activities and information pertaining to biochemical pathways. However, when the intriguing profiles of tumor cell growth inhibition, cytotoxicity, telomerase inhibitory activity and PARP inhibitory activity were initially uncovered, it was found that a molecular target should be cleared: these small molecule-designed telomere-targeted inhibitors exacted antitumor activity and PARP inhibition via unknown mechanisms of action. In this study, we describe the multidisciplinary evidences, approaches and experiments undertaken to elucidate SARs in the PARP inhibitory activity of several anthraquinone-derived compounds as potential antitumor targets. The molecular approach, evidence of antitumor activity and telomerase activity has also been obtained and discussed. As summarized in Table 1, the seven synthetic small molecules were studied for antiproliferative activity against human NSCLC cell lines in the NCI Drug Screen Program. A common structural characteristic of PARP-1 inhibitors is the presence of a carboxamide group attached to a poly-cyclic skeleton. The oxygen atom from the carbonyl group may acts as a hydrogen bond acceptor, and the hydrogen atom from the amide group functions as a hydrogen bond donor in the hydrogen-bond interaction with PARP-1 [45]. It has been recently reported that a newly corn-shaped hydrophobic subsite emerges from the active site of PARP-1 when PARP-1 complex is combined with the PARP-1 inhibitors [47]. Most of the selected test compounds were added to NSCLC lines; perhaps they were at the micro-molar level. All seven selected compounds almost exhibited notable antiproliferative effects on A549 cells, with GI_50_ values in the low range (Table 2). In conjunction with the studies, we planned to use these seven compounds to further investigate of an *in vitro* PARP-1 inhibition assay. Understanding the cell death pathways for selective killing of chemo-resistant NSCLC tumors would lead to more effective strategies. So far, no chemotherapeutic agents have been developed that exploit PARP-1 hyper-activation for NSCLC therapy. It has therefore been proposed that inhibition of PARP-1 may increase the efficacy of DNA-damaging anti-cancer therapy. As noted in these companion small molecules, **NSC747854** is chemically novel and biologically unique in its mechanism of action and *in vitro* pattern of activity. In this research, we present evidence that **NSC747854** possess a number of pharmacologically desirable properties which it has a multi-log differential pattern of activity and unique mechanisms of action in the NCI's 60 cell lines screen (Figure 3).

**Table 1:** Effects of *in vitro* anticancer activity of compounds **NSC746364**, **NSC746365**, **NSC 746366, NSC747515**, **NSC747854**, **NSC749232** and **NSC749235** in the NCI's 9 NSCLC cell lines.

Compound	NSCLC Cell Lines (Inhibition concentration, µM)								
	A549/ATCC	EKVX	HOP-62	HOP-92	NCI-H226	NCI-H23	NCI-H322M	NCI-H460	NCI-H522
**NSC746364**									
GI_50_	1.45	12.8	4.56	10.4	15.5	11.0	1.86	1.37	1.27
TGI	4.43	31.4	20.5	31.7	32.4	28.1	4.91	3.35	13.1
LC_50_	>100	77.4	60.8	96.6	67.4	71.8	83.1	8.20	66.4
**NSC746365**									
GI_50_	1.44	3.05	1.73	0.01	6.56	1.53	2.85	1.90	1.40
TGI	3.95	11.0	5.80	0.06	20.3	3.55	1.59	4.85	4.59
LC_50_	15.5	34.6	24.0	3.48	45.9	8.27	4.71	>100	19.3
**NSC746366**									
GI_50_	2.20	3.57	2.48	0.45	2.72	2.38	1.81	2.69	3.41
TGI	12.7	20.4	11.6	0.82	12.6	17.3	10.9	>100	17.1
LC_50_	74.7	>100	45.4	34.6	58.3	>100	42.0	>100	>100
**NSC747515**									
GI_50_	3.80	11.2	18.0	-	19.3	11.6	8.21	1.68	15.9
TGI	15.0	26.5	33.2	-	36.6	25.7	21.4	4.77	32.5
LC_50_	41.8	62.4	61.2	-	69.7	56.9	47.3	29.3	66.2
**NSC747854**									
GI_50_	3.26	4.20	5.90	-	3.74	5.73	0.72	2.13	1.22
TGI	12.6	20.1	32.3	-	22.1	19.7	20.6	12.8	24.9
LC_50_	38.9	47.9	48.9	-	58.7	46.9	45.7	38.9	50.0
**NSC749232**									
GI_50_	12.3	1.98	2.97	9.73	8.81	1.78	3.76	5.81	8.95
TGI	29.2	14.9	9.49	31.7	20.8	8.28	13.7	14.1	2.03
LC_50_	>100	>100	24.4	>100	49.1	31.4	45.4	34.1	-
**NSC747235**									
GI_50_	7.58	6.69	19.4	7.97	8.32	10.7	8.70	3.79	13.8
TGI	24.3	28.8	30.5	91.8	31.1	30.1	43.3	15.2	29.0
LC_50_	67.2	>100	95.4	>100	>100	84.8	>100	45.1	61.0

**Table 2:** Cytotoxicity of selected seven compounds in NSCLC cell lines of the NCI *in vitro* 60-cell lines Drug Screen Program were on the micro-molar level.

NSCL Cancer Cell Lines	-Log GI_50_								
	A549/ATCC	EKVX	HOP-62	HOP-92	NCI-H226	NCI-H23	NCI-H322M	NCI-H460	NCI-H522
**NSC746364**	5.84	4.89	5.34	4.98	4.81	4.96	5.73	5.86	5.9
**NSC746365**	5.84	5.52	5.76	7.83	5.18	5.82	5.55	5.72	5.86
**NSC746366**	5.66	5.45	5.6	6.35	5.57	5.62	5.74	5.57	5.47
**NSC747515**	5.42	4.95	4.74	---	4.71	4.94	5.09	5.77	4.8
**NSC747854**	5.49	5.38	5.23	---	5.43	5.24	6.14	5.67	4.91
**NSC749232**	4.91	5.7	5.53	5.01	5.05	5.75	5.42	5.24	6.05
**NSC749235**	5.12	5.17	5.03	5.1	5.08	4.97	5.06	5.42	4.86

The better cytotoxicity of each compound against nine cells was highlighted.

### In vitro cytotoxicity evaluation

**Figure 4:**
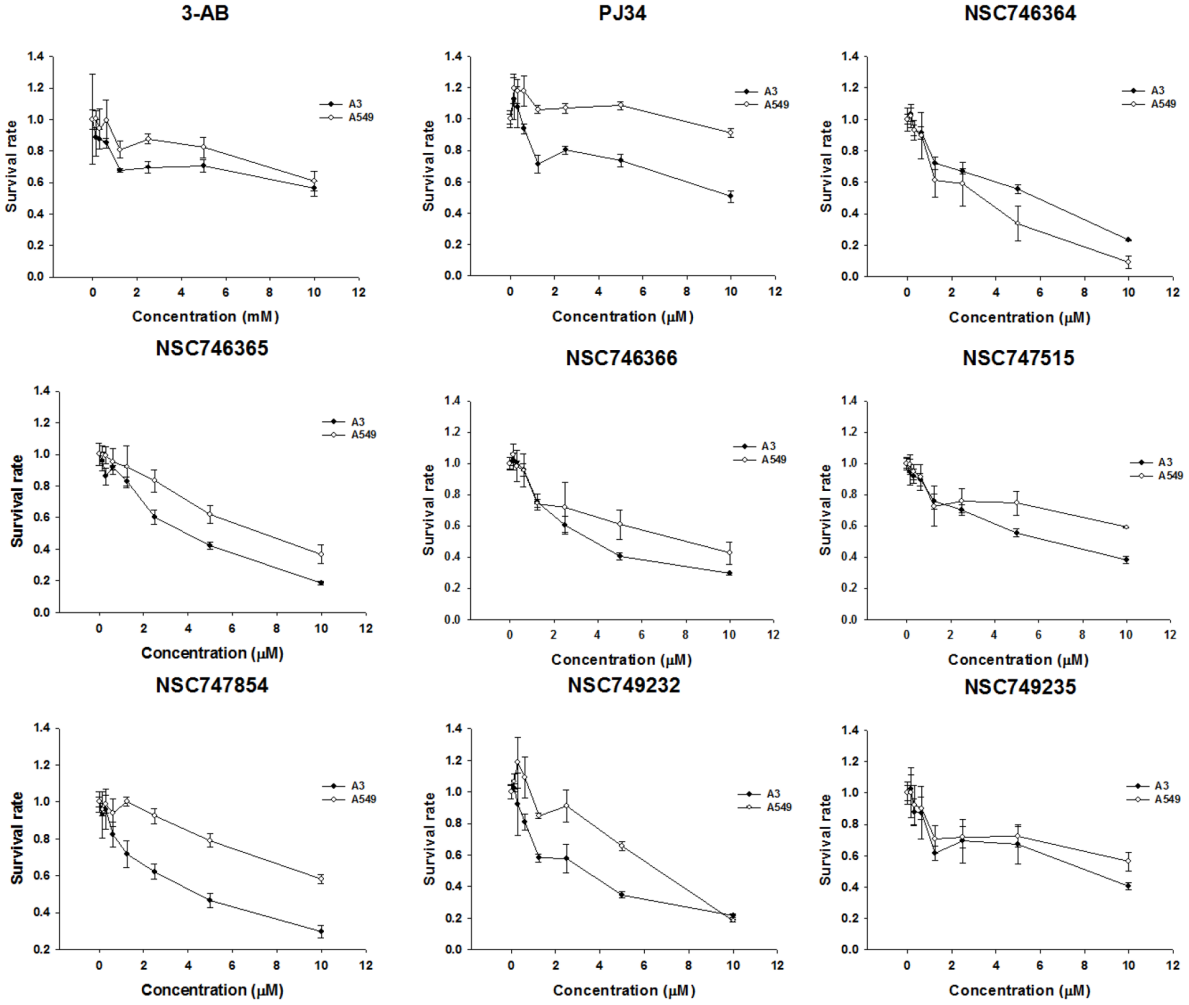
Dose-response effects of selected seven compounds on cell growth of A549 and A3 cells were treated with various concentrations of testing compounds for 48 h, and cell viability was determined by the MTT assay. Cell viability values are expressed relative to those wells in which various compounds were not added (100% control value). Each point represents a mean values ± SD of at least three independent experiments.

**Figure 5:**
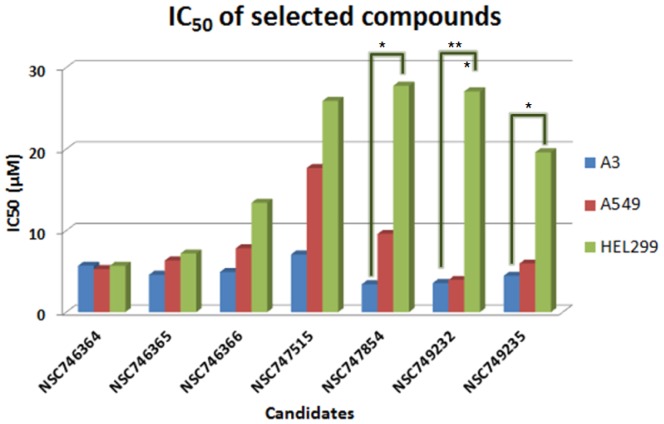
The survival rate in A3, A549, and HEL299 cell line treated with putative PARP-1 inhibitor candidates and well-known PARP-1 inhibitors (* p<0.05; **p<0.01). Values represent an average of at least three independent experiments.

**Figure 6:**
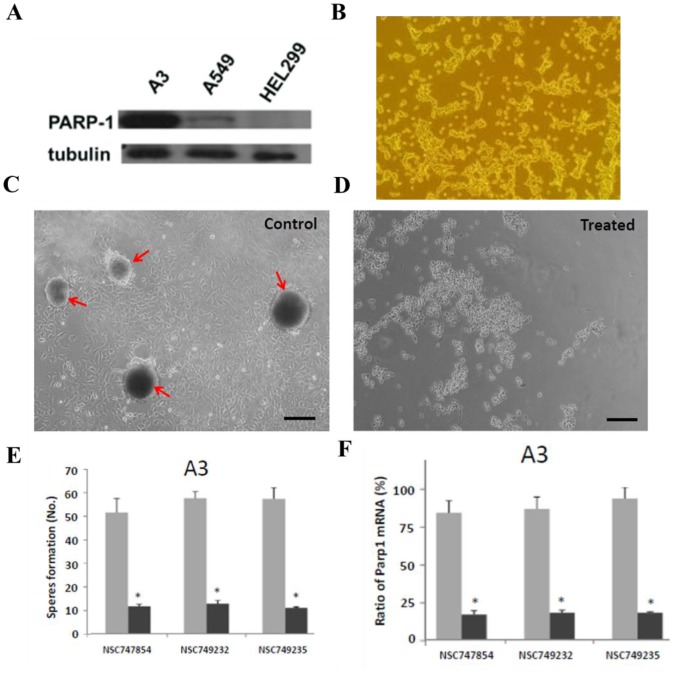
In vitro assay for evaluating the effects of drug treatment on A3 CSC-like clone. a) When compared to the expression of a housekeeping protein β-tubulin, the extent of PARP-1 in A3 cells were estimated to be more than in A549 and HEL299 cells. b) The morphology of A3 cells cultured on 10% serum-contained medium. c) Under serum-free with bFGF and EGF (10 ng/ml) culture, the tumor sphere formation (arrows) of A3 cells was observed (bar = 60 µm). The compounds of NSC747854, NSC749232, and NSC749235 decreased d) the cell viability, e) block the sphere formation, and f) inhibit the mRNA expression levels of Parp1 in treated A3 cells. Each point represents a mean values ± SD of at least three independent experiments.

For the further and detailed evaluation of these seven compounds, we use MTT assay upon A549 cells to compare these compounds to 3-AB and PJ-34 which the data are shown in Figure 4. The IC_50_ of four compounds (**NSC746364**, **NSC746365**, **NSC746366** and **NSC749232**) were between 1 and 10 µM in A549 cells. Interestingly, the cytotoxicity of seven compounds to the A3 cell line was better than it was to the A549 cell line. In general, both of the two drugs, 3-AB and PJ-34, show worse cytotoxicity than the selected compounds (Table 3). Compared to the A549 cell line, we found out that the amounts of PARP-1 expression were markedly increased in the A3 cell line in Figure 6A. Thus, we took the A3 cell line to be our cell line-based drug screening platform. The results indicated that A3 cells to six compounds (except **NSC746364**) were more sensitive than A549 cells. This interesting phenomenon also appeared in PARP-1 inhibitors (3-AB and PJ-34) (Table 3). Base on a series of cases of this kind of research, we hoped that the development of new drugs not only promises to furnish good cytotoxicity, but also promises to result in no significant influence on normal cells. Therefore, we used HEL299 cells as our normal cell lines; we treated this normal cell line with seven compounds and two PARP-1 inhibitors in order to test the cytotoxicity of these selected compounds and two PARP-1 inhibitors upon normal cells. The IC_50_ data of MTT assays of these compounds are shown in Figure 5. The cytotoxic effects of seven compounds were slightly better in Oct-4- and Nanog-overexpressed A549 clone (A3) and cancer cells than in normal cells. Our previous study had established Oct-4- and Nanog-overexpressed A549 clone (clone #3; A3), and this A3 adenocarcinomal cell line presented the properties of cancer stem-like cells and epithelial-mesenchymal transdifferentiation as well as metastatic ability [46], [48]. The results of the western blot demonstrated that this A3 cell line was highly expressed PARP-1, but not in parental the A549 and the HEL299 cells (Figure. 6B). Importantly, Furthermore, A3–A549 cell line endogenously presents the high expression levels of CD133 (cancer stem cell marker) and ABCB1 as well as ABCG5 (drug-resistant genes), as compared to parental A549 cells [46], [48]. In this study, the results of the western blot demonstrated that this A3 cell line was highly expressed PARP-1, but not in parental the A549 and the HEL299 cells (Figure 6). In Figure 6c, the data showed that A3 clone can significantly form the tumor-spheres in the serum free medium with bFGF and EGF (10 ng/ml). Moreover, the results of sphere formation and cell viability assay demonstrated that the compounds of **NSC747854**, **NSC749232**, and **NSC749235** in treated A3 clone can effectively block the sphere formation and inhibit the cell growth in vitro (Figure 6D and 6E). Notably, using quantitative RT-PCR, we demonstrated that the compounds of **NSC747854**, **NSC749232**, and **NSC749235** can inhibit the expression levels of PARP-1 in treated A3 cells (Figure 6F). After A3, A549 and HEL299 cells were treated with compounds, we compared the survival rate respectively between the A549 cell line and the HEL299 cell line by use of an unpaired two-tailed t-test which **NSC749232** had a significant difference. Compared to the A3 cell line, **NSC747854**, **NSC749232** and **NSC749235** had a significantly different survival rate to the HEL299 cell line. Noticeably, 3-AB and PJ-34 would also be relatively harmful to normal fibroblast cell line (HEL299) in this study. The results imply that **NSC747854** does not affect the growth and general transcription of normal cells. Additionally, **NSC747854** and other selected anthraquinone-derived small molecules are in general more potent than 3-AB and PJ-34. It was first reported that anthraquinone analogues are potent human PARP inhibitors. Together, the data presented here expand our insight into the PARP inhibitors and support the resource-demanding lead optimization of structurally related small molecules for human cancer therapy. The IC_50_ data of MTT assays of these compounds are shown in Figure 5.

**Table 3:** The IC_50_ in A3, A549, and HEL299 cell line.

Compounds	IC_50_ (µM)^a^		
	A3	A549	HEL299
**NSC746364**	5.90	4.49	5.67
**NSC746365**	5.21	7.66	7.19
**NSC746366**	5.77	7.76	13.41
**NSC746515**	7.05	11.80	25.90
**NSC747854**	5.88	12.20	27.80
**NSC749232**	4.85	6.62	27.10
**NSC749235**	7.67	10.6	19.60
**3-AB**	10.80	13.10	5.98
**PJ-34**	9.44	31.70	14.70

Values represent an average of at least three independent experiments.

a IC_50_, drug concentration inhibiting 50% of cellular growth following 48 h of drug exposure.

Also, the current work illustrates that the IC_50_ of these selected seven compounds against two cancer cells were smaller than that of the PARP-1 inhibitor. Most of these selected compounds were devoid of any toxicity towards normal fibroblasts. To elucidate the SARs and *in vitro* anticancer activity, we correlated their activity profile (GI_50_, TGI and LC_50_) in the screening system and also their effects on cell proliferations, cytotoxicity and PARP-1 inhibitory effects.

### PARP inhibitory evaluation of the selected small molecules

To date, a number of families of compounds have been developed and their PARP activity and cytotoxicity have been extensively studied. Taken together, these results suggested that the anti-cancer potentials of anthraquinone-derived small molecules against cell-based are, at least in part, ascribed to its anti-cancer effects and PARP inhibitory activity. The unique molecular characterization, cytotoxicity and telomerase activity profiles warrant further investigation and indicate a potential novel mechanism of anticancer action involved. Small molecules that modulate PARP-1 inhibitory will likely provide new insights into the regulation of this key developmental pathway and ultimately provide our field with potential new pharmacological agents, such as anti-cancer drugs. Although many drug discovery programs screen for novel small-molecule PARP-1 inhibitors, no appropriate candidate has yet been found to possess enough potent and specific inhibitors. As a result, we found that dose-dependent potential PARP-1 inhibitor **NSC747854** was the only compound which followed all of the criteria mentioned above.

**Figure 7:**
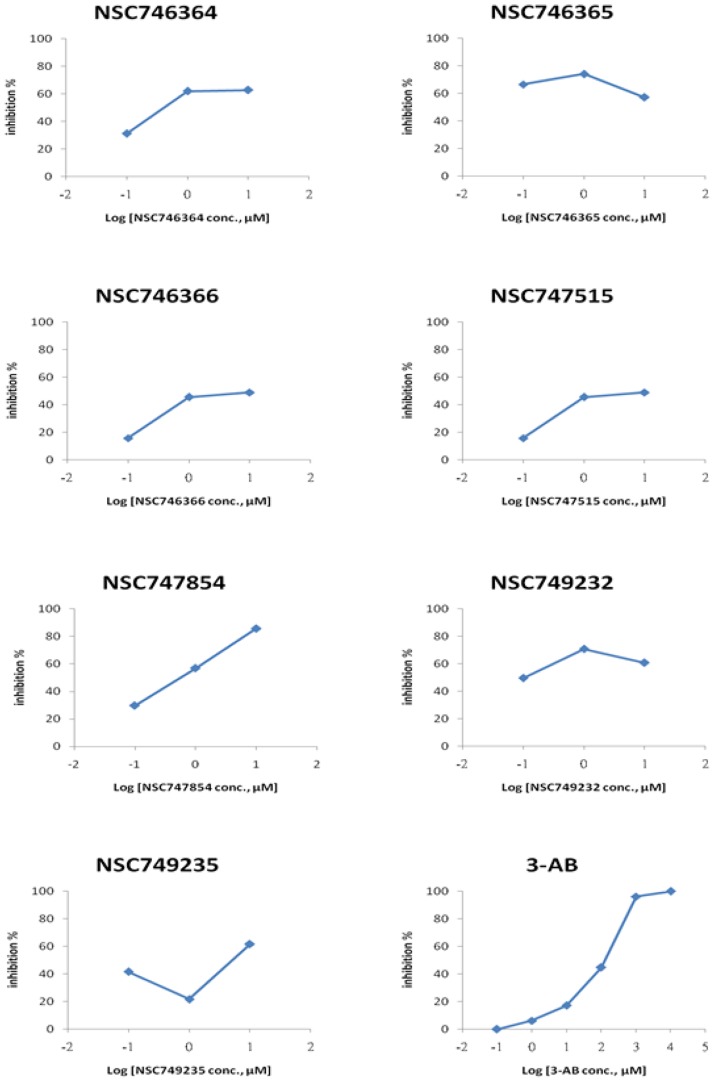
The results of PAPR inhibitory percentage in seven putative PARP-1 inhibitor candidates and PARP-1 inhibitor (3-AB). 3-AB had dose-dependent PARP inhibitory effects, and the most obvious dose-dependent PARP inhibitory effect in the seven PARP inhibitor candidates was **NSC747854**. At 0.1 and 1 µM, PARP inhibitory effect of **NSC746364**, **NSC746366** and **NSC749232** increased along with the concentration increased, but at 1 and 10 µM, PARP inhibitory effect had no significant difference.

We used histone-coated 96-well plate treated with seven putative PARP-1 inhibitors or well-known PARP-1 inhibitors (3-AB), and then added PARP enzyme to each well. PARP enzyme catalyzes the NAD-dependent addition of poly (ADP-ribose) to histone, and diluted Strp-HRP could bind to PARylation histone. TACS-Sapphire™ yielded blue under the HRP existence. In order to systematically evaluate the potential anticancer activity, the compounds were tested for their cytotoxicity *in vitro* against 60 human cancer lines in the NCI anticancer drug screen as well as for dose response curves and telomerase activity. Cell growth was analyzed by the MTT assay, with differences between dose-response curves analyzed non-parametrically. Telomerase activity was detected by a modified version of the PCR-based assay and TRAP assay [31]. **NSC746364**, **NSC746365** and **NSC746366** are structurally 2,7- diamidoanthraquinone derivatives; **NSC747515**, **NSC747854** and **NSC749235** are 1,2-heteroannelated anthraquinone derivatives, **NSC749232** is an asymmetrical 1,2-diamidoanthraquinone derivative, compared with other clinically used anticancer agents and have exhibited a unique cytotoxicity at the PARP-1 high-expressed cell line, A3. Since PARP is an important component of cancers, we are interested in examining the effects of these compounds on PARP activity, so we revealed a potential PARP inhibitor through various test approaches. As shown in Figure 7, 3-AB, the PARP inhibitory effect behaved in a dose-dependent manner. The PARP inhibitory effects of seven compounds revealed that **NSC746364**, **NSC746366** and **NSC749232** inhibit function of PARP enzyme increase along with the concentration increase at 0.1 and 1 µM. However, PARP inhibitory effects had no difference at 1 and 10 µM. As described above, **NSC746364**, **NSC746366** and **NSC749232** show dose-dependency only at low concentrations. **NSC746364** and **NSC746366** showed a PARP inhibitory effect of 56% and 47%, respectively. **NSC747515** had a similar PARP inhibitory effect at 0.1, 1 and 10 µM. **NSC747854** had dose-dependent PARP inhibitory manners at three different concentrations, and reached the maximum inhibitory effect of 86% at 10 µM (Table 4). Pharmacodynamic studies of PARP-1 inhibitor, such as 3-AB, showed dose-dependent inhibition of PARP activity in peripheral blood mononuclear cells. Toward this end, **NSC747854** showed the maximum inhibitory effect in a dose-dependent PARP inhibitory manner. Hence, **NSC747854** may be the most potent PARP inhibitor candidate which followed all of the criteria mentioned above. In this investigation, we continued to focus our attention on the role of **NSC747854** and to understand the basis of pharmacophore selectivity. Of particular note is the significant difference with these selected compounds, compared with that of 3-AB and PJ-34. Interestingly, most of these selected compounds contain a unique amine with one to three carbon space linker moieties which could provide a structural basis for the differences in potency between these and other PARP-1 inhibitors. From a general structure-activity standpoint, tricyclic planar anthraquinone demonstrated that the structural variations could result in significant changes in specificity and potency with regard to anticancer activity. An electron-rich aromatic ring system, including a carboxamide group, should have at least a hydrogen atom on the amide nitrogen [47]. The amidinium moiety is known to contribute to the stabilization of DNA recognition element through electrostatic and hydrogen bonding interactions [49]. Therefore, hydrogen bonds are frequently used as recognition elements due to their directionality and also due to being attractive factors to the biological activity (Figure 1) [50]. Many classes of drugs have substantial curvature, high DNA affinity, binding to the minor-groove or major-groove, interference with DNA-associated enzymes (e.g. telomerase), and Poly (ADP-ribose) polymerase, PARP. The binding affinities and specificities observed suggest that the incorporation of a variety of moieties will lead to substances that interact with PARP targets. This approach greatly expands the utility of the substituents and related pharmacophore for the construction of drugs in general. Therefore, it is desirable to design PARP inhibitors in accordance with various anticancer parameters. It was first reported that anthraquinone analogues are potent human PARP inhibitors.

**Table 4:** Inhibition of PARP activity by selected seven compounds whose tested concentrations were 0.1 µM, 1 µM, and 10 µM.

	PARP inhibitory activity (%)		
	0.1 (µM)	1 (µM)	10 (µM)
**NSC746364**	31	54	56
**NSC746365**	50	56	51
**NSC746366**	16	45	47
**NSC746515**	20	22	24
**NSC747854**	30	57	65
**NSC749232**	42	51	52
**NSC749235**	40	28	58
**3-AB**	4	10	18

After showing hopeful results in multiple preclinical studies, two benzo[c]phenanthridine derivatives: dihydronitidine (DHN), nitidine (NTD) have been shown selective cytotoxicity [51], [52]. PJ-34, was the most promising small molecule and also efficiently prevented the development of MDA-231 and lung cancer cells H1299 without inducing detectable toxic effects in the normal human proliferative cells [23]. We could know that phenanthrene-related derivatives play an important role in the field of PARP inhibitors and select at human cancer cells. In conclusion, the data in this article indicate that the compounds exhibit potent and differential *in vitro* activity against NSCLC cells and especially PARP-1 high expressed cancer cells compared with normal fibroblast cells in the course of these experiments. On the basis of these pharmacologically desirable properties and the broad spectrum *in vitro* index of activity (GI_50_, TGI, and LC_50_) reported, **NSC747854** is currently undergoing more detailed preclinical pharmacology and toxicology studies through our laboratory. Collectively, these studies show that anthraquinone-derived small molecules can induce pharmacological and biochemical features of PARP-1 inhibition. Our data clearly extends the biological evidences used by the seven selected compounds. As far as we know, anthraquinones have never been reported to cause PARP-1 inhibition. On the whole, our studies support the conclusion that the tested molecules **NSC747854** shared a common target in telomerase and PARP that may be a novel less toxic modality of cancer therapy. PARP-1 inhibitor's use in treating cancer is a high-profile issue today. High-expressed PARP-1 is an import-resistant mechanism. In our study, we found out that there is a high PARP-1 expression in the A3 cell line, which could also be exploited for drug screening of PARP-1 inhibitors. As a part of our continuing search for novel, potent and cell-permeable inhibitors of this enzyme, additional anthraquinone-derived analogues of **NSC747854** were also explored. Preliminary mechanistic studies, including the influence on PARP-1 inhibition pathways and cytotoxicity, were thus performed in order to reveal a more detailed picture on the possible targets. Further evaluation will be reported in the near future.

## Materials and Methods

### Design and synthesis of chemical compounds

We previously reported some of the potential of novel small molecules as anticancer agents [17]–[26]. The details of synthesis and chemical characterization of **NSC746364**, **NSC746365**, **NSC746366, NSC747515, NSC747854, NSC749235**, and **NSC749232** have been described previously in a separate report and so have some new synthetic derivatives from our laboratory. Compounds such as those described here, which interact selectively with NCI Developmental Therapeutics Program's *in vitro* 60 cell lines screen and inhibit telomerase, are potentially useful as inhibitors of the proliferation of cells that require telomerase to maintain telomere length for continued growth.

### Evaluation of anthraquinone-derived small molecules against NCI-60 panel tumor cell lines in vitro and summary of preliminary study

As a primary screening, seven selected compounds were submitted to the National Cancer Institute (NCI) cell lines screen for evaluation of their anticancer activity [53]. From the data analysis, it follows that approximately 95% of the actives from the 60 cell lines screen can be identified. The detailed methods used for the 60 cell lines panel have been described elsewhere [54]–[59]. Briefly, cellular protein levels were determined after 48 hours of drug exposure by SRB colorimetry. Through the use of a time zero cell control, the cell growth can be determined for each cell line thus allowing calculations of GI_50_, TGI, and LC_50_. Comparison to plates not exposed to drug permits determination of concentration and times of exposure conferring GI_50_, TGI, and LC_50_. These data are then plotted as mean bar graphs and as dose-response curves. By these criteria, seven compounds reported from our compounds bank were all active and passed on for evaluation in the full panel of 60 human tumor cell lines. The panel is organized into nine subpanels representing diverse histology: leukemia, melanoma, and cancers of the lung, colon, kidney, ovary, breast, prostate, and central nervous system.

### Cell culture

Non-small cell lung cancer cells A549 and Oct-4- and Nanog-overexpressed A549 clone (clone #3; A3), presented the properties of cancer stem-like cells, as well as epithelial-mesenchymal transdifferentiation, according to our previous study [46]. They were grown in the same conditions as RPMI 1640 media supplemented with 10% fetal bovine serum (FBS), 100 units/mL penicillin, 100 mg/mL streptomycin, 1 mM sodium pyruvate, 2 mM l-glutamine and 1% non-essential amino acid (NEAA) in a humidified atmosphere with 5% CO_2_ at 37°C. Normal fibroblast cells HEL299 from American Type Culture Collection (ATCC) were grown in MEM media supplemented with 10% FBS, 100 units/mL penicillin, 100 mg/mL streptomycin, 1 mM sodium pyruvate, 2 mM l-glutamine and 1% NEAA, in a humidified atmosphere with 5% CO_2_ at 37°C.

### MTT cytotoxicity assay

The tetrazolium reagent (MTT; 3-(4,5-dimethylthiazol-2-yl)-2,5-diphenyl- tetrazolium bromide, USB) was designed to yield a colored formazan upon metabolic reduction by viable cells [60], [61]. Approximately 5×10^4^ cells were plated onto each well of a 24-well plate and incubated in 5% CO_2_ at 37°C for 24 hours. To assess the *in vitro* cytotoxicity, each compound was dissolved in DMSO and prepared immediately before the experiments and then was diluted into the complete medium before being added to cell cultures. Test compounds were then added to the culture medium in various designated concentrations. After 48 hours, an amount of 100 µl of MTT was added to each well, and the samples were incubated at 37°C for 2 h. 100 µl DMSO was added to each well. The absorbency at 560 nm and reference absorbency at 670 nm were measured using an ELISA reader.

### PARP inhibitory assay

Strip wells were removed from the wrapper and 50 µl of 1X PARP Buffer was added to each well to rehydrate the histones. They were incubated at room temperature for 30 minutes. The 1X PARP buffer was removed from the wells by tapping the strip wells on paper towels. The serial dilutions of inhibitor of interest were added to appropriate wells. The diluted PARP enzyme (0.5 unit/well) was then added to the wells containing inhibitor. They were incubated for 10 minutes at room temperature. The PARP Enzyme-High Specific Activity (PARP-HAS) standard was serially diluted in cold micro-tubes with 1X PARP buffer such that the total activity was 1 unit/25 µl, 0.5 units/25 µl, 0.25 units/25 µl, 0.1 units/25 µl, 0.05 units/25 µl, 0.025 units/25 µl, and 0.01 units/25 µl, respectively. The 25 µl of each standard was added to triplicate wells. The 25 µl of 1X PARP cocktail were distributed into each well by using a multichannel pipettor. The final reaction volume is 50 µl. They were incubated along with the strip wells at room temperature for 60 minutes. The strip wells were washed 2 times with 1X PBS +0.1% Triton X-100 (200 µl/well) followed by 2 washes with 1X PBS. All the liquid was removed following each wash by tapping strip wells onto paper towels. The 50 µl diluted Strep-HRP were added per well. They were incubated at room temperature for 60 minutes. The strip wells were washed 2 times with 1X PBS +0.1% Triton X-100 (200 µl/well) followed by 2 washes with 1X PBS. All the liquid was removed following each wash by tapping strip wells onto paper towels. The 50 µl pre-warmed TACS-Sapphire™ colorimetric substrate was added per well and incubated, in the dark, for 15 minutes at room temperature. The reactions were stopped by adding 50 µl per well of 0.2 M HCl. The absorbance was read at a wavelength of 450 nm.

### Western blot assay in A3, A549 and HEL299 cells

For immunoblot analysis, cells were harvested in lysis buffer. The resulting lysates were electrophoresed in a 10% sodium dodecyl sulfate polyacrylamide gel and transferred onto polyvinylidenedifluoride (PVDF) membrane (Millipore, Bedford, MA). The membranes were blocked with Tris-buffered saline with Tween 20 (10 mM Tris-HCl, pH 7.4, 150 mM NaCl, 0.1% Tween 20) containing 5% milk and probed with poly (ADP-ribose) polymerase-1 (PARP-1). Immunoactivity was visualized by the enhanced hemiluminescence's detection system (ECL, Amersham Biosciences, Pittsburgh, PA, USA) in accordance with the manufacturer's instructions.

### RNA isolation and quantitative polymerase chain reaction, qPCR

RNA was isolated from cells with the TRIzol (Invitrogen) reagent (Roche), and this was followed by RNA clean up with the RNeasy Minikit (QIAGEN). cDNA was produced with the First Strand cDNA Synthesis Kit (Roche). Real-time quantitative PCR reactions were set up in triplicate with the Brilliant II SYBR Green QPCR Master Mix (Stratagene) and run on an Mx3000P QPCR System (Stratagene).

### Statistical analysis

Data are presented as means ± SD for at least three independent experiments. Comparisons between groups for all tests were done using an unpaired two-tailed t-test; the Generalized Estimating equation (SPSS Advanced Statistics 17.0) was used for estimating cell viability. A *P* value of less than 0.05 was considered statistically significant.
